# Integrated modeling and experimental approach for determining transcription factor profiles from fluorescent reporter data

**DOI:** 10.1186/1752-0509-2-64

**Published:** 2008-07-17

**Authors:** Zuyi Huang, Fatih Senocak, Arul Jayaraman, Juergen Hahn

**Affiliations:** 1 Artie McFerrin Department of Chemical Engineering, Texas A&M University, College Station, TX 77843-3122, USA

## Abstract

**Background:**

The development of quantitative models of signal transduction, as well as parameter estimation to improve existing models, depends on the ability to obtain quantitative information about various proteins that are part of the signaling pathway. However, commonly-used measurement techniques such as Western blots and mobility shift assays provide only qualitative or semi-quantitative data which cannot be used for estimating parameters. Thus there is a clear need for techniques that enable quantitative determination of signal transduction intermediates.

**Results:**

This paper presents an integrated modeling and experimental approach for quantitatively determining transcription factor profiles from green fluorescent protein (GFP) reporter data. The technique consists of three steps: (1) creating data sets for green fluorescent reporter systems upon stimulation, (2) analyzing the fluorescence images to determine fluorescence intensity profiles using principal component analysis (PCA) and K-means clustering, and (3) computing the transcription factor concentration from the fluorescence intensity profiles by inverting a model describing transcription, translation, and activation of green fluorescent proteins.

We have used this technique to quantitatively characterize activation of the transcription factor NF-κB by the cytokine TNF-α. In addition, we have applied the quantitative NF-κB profiles obtained from our technique to develop a model for TNF-α signal transduction where the parameters were estimated from the obtained data.

**Conclusion:**

The technique presented here for computing transcription factor profiles from fluorescence microscopy images of reporter cells generated quantitative data on the magnitude and dynamics of NF-κB activation by TNF-α. The obtained results are in good agreement with qualitative descriptions of NF-κB activation as well as semi-quantitative experimental data from the literature. The profiles computed from the experimental data have been used to re-estimate parameters for a NF-κB model and the results of additional experiments are predicted very well by the model with the new parameter values. While the presented approach has been applied to NF-κB and TNF-α signaling, it can be used to determine the profile of any transcription factor as long as GFP reporter fluorescent profiles are available.

## Background

Systems Biology seeks to develop models for describing cellular behavior on the basis of regulatory molecules such as transcription factors and signaling kinases. The control of gene expression by transcription factors is an integral component of cell signaling and gene expression regulation [1,2]. Different transcription factors exhibit different expression and activation dynamics, and together govern the expression of specific genes and cellular phenotypes [3]. An important requirement for the development of these signal transduction models is the ability to quantitatively describe the activation dynamics of transcriptions so that parameters can be estimated for model development. The activation of transcription factors under different conditions have been conventionally monitored using protein binding techniques such as electrophoretic mobility shift assay or chromatin immunoprecipitation [4]. While these techniques provide snapshots of activation at a small set of single time points, they can yield only qualitative or semi-quantitative data at best. This approach also requires the use of multiple cell populations for each time point at which transcription factor activation is to be measured, and often, the true dynamics of transcription factors are not captured due to limited sampling points and frequencies. Hence, these methods are not ideal for investigating time-dependent activation of transcription factors in a quantitative manner.

**Figure 1:**
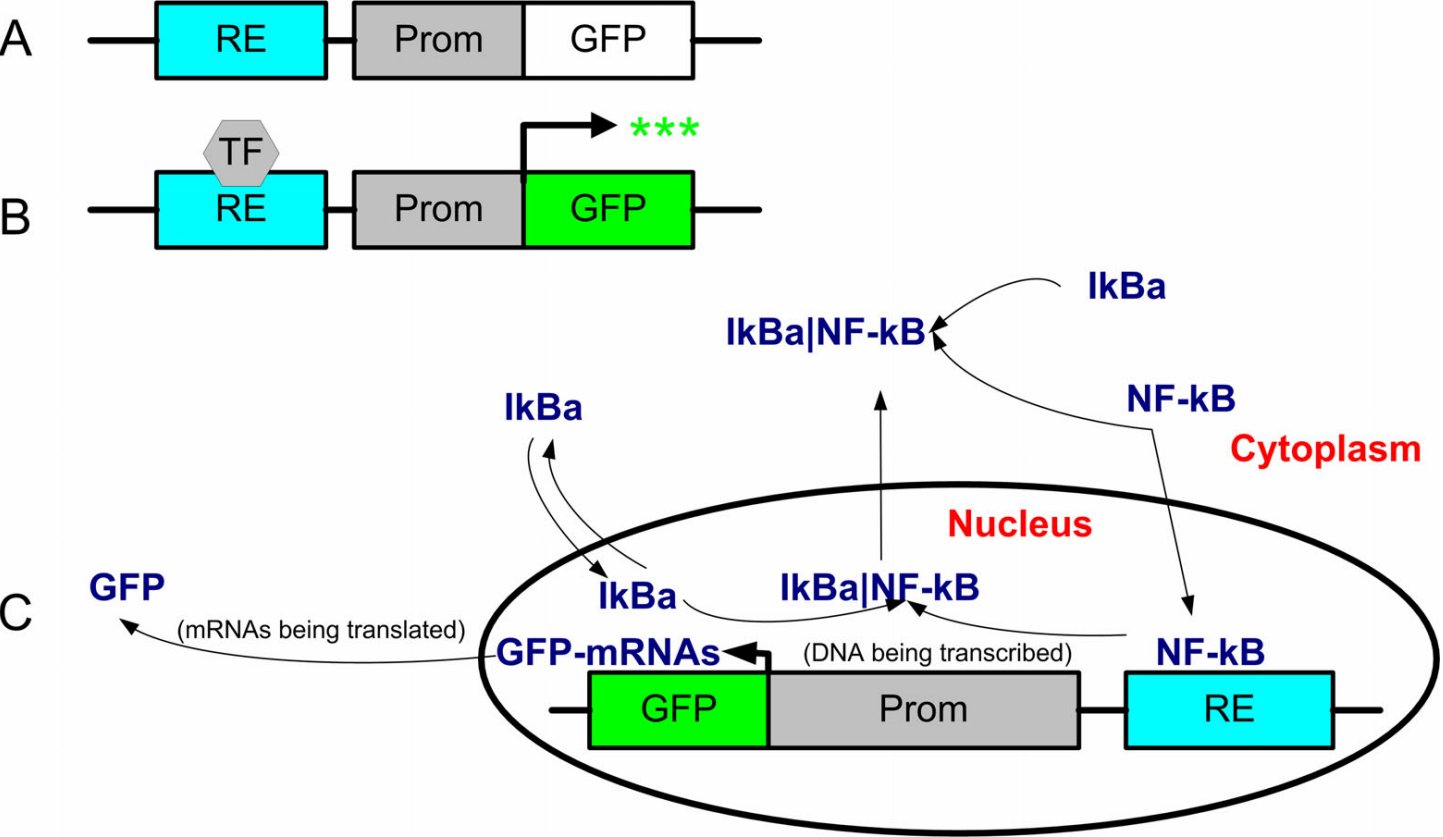
**GFP-based reporter systems for investigating transcription factor (TF) activation.** The DNA response element (RE) to which the TF binds is upstream of a minimal promoter that controls GFP expression (A) No fluorescence is observed in the absence of TF binding, (B) Binding of TF leads to promoter activation and GFP fluorescence, (C) Dynamics of a TF (e.g., NF-κB) is the sum of activation of the TF and the dynamics of GFP expression.

More recently, fluorescence-based reporter systems have been developed for the continuous and non-invasive monitoring of transcription factors and the elucidation of regulatory molecule dynamics. Recent studies [5-8] have used green fluorescent protein (GFP) as a reporter molecule for continuously monitoring activation of a panel of transcription factors, underlying the inflammatory response in hepatocytes for 24 h. These systems involve expressing GFP under the control of a minimal promoter such that GFP expression and fluorescence is observed only when a transcription factor is activated (i.e., when the transcription factor binds to its specific DNA binding sequence and induces expression from a minimal promoter) (Figure 1A &1B). The dynamics of GFP fluorescence is used as the indicator for dynamics of the transcription factor being profiled. The primary drawback with this approach is that it does not provide direct activation rates of the transcription factors being investigated. Even though transcription factor dynamics influence GFP dynamics, the relationship between the two is non-trivial as the induction of GFP fluorescence itself involves multiple steps (i.e., transcription of GFP mRNA, GFP protein translation, post-translational processing, etc) [9], and not all of these steps contribute equally to regulation of GFP expression. The observed fluorescence dynamics in GFP reporter cell systems is the result of two different dynamics: (i) the dynamics of transcription factor activation by a soluble stimulus-mediated signal transduction pathway and (ii) the dynamics of GFP expression, folding, and maturation. Therefore, it is necessary to uncouple the effects of these independent systems in order to quantitatively determine transcription factor activation profiles underlying cellular phenotypes.

In this study, we use an integrated modeling and experimental strategy for deriving transcription factor activation rates from GFP-based fluorescent reporter systems. Using GFP reporter data for the activation of the transcription factor NF-κB by the cytokine TNF-α, (Figure 1C), we demonstrate that NF-κB activation dynamics can be accurately determined from GFP reporter profiles. The quantitative data that is determined from the presented approach can be used to update models of signal transduction pathways. This is illustrated by first developing a model describing TNF-α signal transduction based upon the models presented by Rangamani and Sirovich [10] and Lipniacki et al. [11] and then re-estimating model parameters. In a final modeling step, the most important parameters of the model are estimated from the data obtained in this work. The presented approach is not limited to NF-κB and can be used to determine the activation profile of any transcription factor as long as GFP reporter fluorescent profiles are available.

## Methods

### Reagents

All cell culture reagents including, Dulbecco's modified Eagle's Medium (DMEM, 4.5 g/L glucose), Bovine serum (BS) were purchased from Hyclone (Logan, UT). Human insulin and penicillin/streptomycin were purchased from Sigma (St. Louis, MO).

### Cell culture

The generation of a NF-κB reporter cell line has been described earlier [5]. Briefly, a reporter plasmid containing 4 tandem repeats of the NF-κB DNA binding sequence upstream of the CMV-minimal promoter and a 2 h half-life variant of the enhanced green fluorescence protein (d2EGFP) was stably introduced into H35 rat liver hepatoma cells by electroporation and selected based on neomycin resistance. Reporter cells were grown in DMEM supplemented with 10% v/v BS, penicillin (200 U/ml), and streptomycin (200 μg/ml).

### Reporter gene assays

H35-NF-κB cells were grown in 6-well tissue culture dishes (Corning, NY) to ~70% confluence prior to the experiment. Reporter cells were stimulated for 30 minutes, 2 hours, and 4 hours or continuously with either 10 ng/mL or 25 ng/ml TNF-α (R&D Systems). All experiments were run in triplicate.

### Fluorescence microscopy

GFP measurements were made using a Axiovert 200 M fluorescence microscope (Zeiss, Thornwood, NY). Cell culture dishes were placed in a controlled environment chamber in the microscope and maintained at 37°C and 10% CO_2 _throughout the experiment. Multiple imaging locations (3 per culture well) were randomly selected and the positions marked before the addition of TNF-α using the 'mark and find' feature of the using the Zeiss AxioVision imaging software. Fluorescence and phase contrast images were obtained at the marked positions throughout the duration of the experiment using a 20X objective every hour for 16 h using an AxioCam MrM digital camera.

### Image Analysis

The series of images taken by fluorescence microscopy were analyzed to generate a time series of data representing the average fluorescence intensity of the cells in the images. In order to compute a fluorescence intensity profile it is required to first determine the areas in the image representing cells where fluorescence can be seen. The procedure for determining these areas makes use of principal component analysis (PCA) and K-means clustering. A second step involves computing the average fluorescence intensity over these areas. The detailed steps involved in these procedures are described in the following. Each RGB image can be represented as a three-dimensional tensor

(1) $$\begin{array}{l} M_{1}=\left[\begin{array}{ccc} r_{11} & \cdots & r_{1m}\\ \vdots & \ddots & \vdots \\ r_{n1} & \cdots & r_{nm} \end{array}\right],M\in {ℛ}^{n,m,3}\\ M_{2}=\left[\begin{array}{ccc} g_{11} & \cdots & g_{1m}\\ \vdots & \ddots & \vdots \\ g_{n1} & \cdots & g_{nm} \end{array}\right]\\ M_{3}=\left[\begin{array}{ccc} b_{11} & \cdots & b_{1m}\\ \vdots & \ddots & \vdots \\ b_{n1} & \cdots & b_{nm} \end{array}\right] \end{array}$$

where the first two dimensions of *M*(*i*,*j*,*k*) refer to the position of a particular pixel on the image, i.e., the *i*-th row and *j*-th column, and the third dimension refers to the red (*k *= 1), green (*k *= 2), or blue (*k *= 3) value of the pixel. It is required to transform this three dimensional tensor, *M*, to a two-dimensional matrix, *X*:

(2) $$X=\left[\begin{array}{ccc} r_{11} & g_{11} & b_{11}\\ \vdots & \vdots & \vdots \\ r_{1m} & g_{1m} & b_{1m}\\ r_{21} & g_{21} & b_{21}\\ \vdots & \vdots & \vdots \\ r_{2m} & g_{2m} & b_{2m}\\ \vdots & \vdots & \vdots \\ r_{n1} & g_{n1} & b_{n1}\\ \vdots & \vdots & \vdots \\ r_{nm} & g_{nm} & b_{nm} \end{array}\right]$$

**Figure 2:**
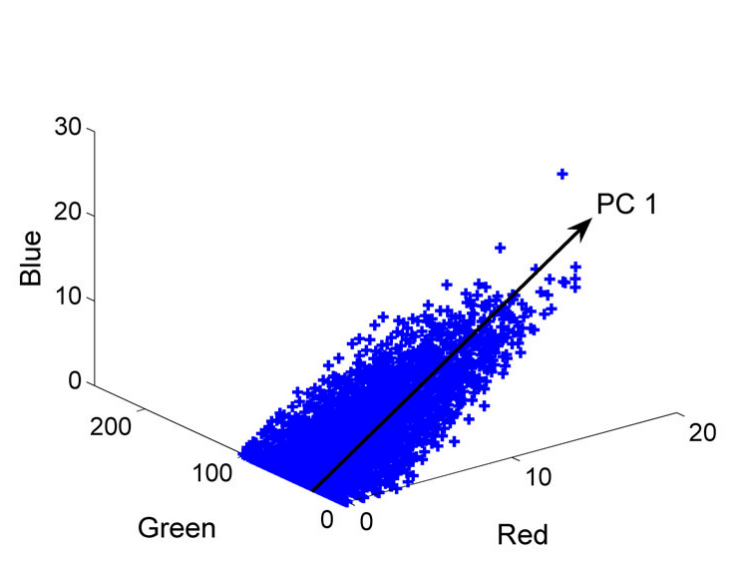
Principal component analysis of fluorescence image data.

**Figure 3:**
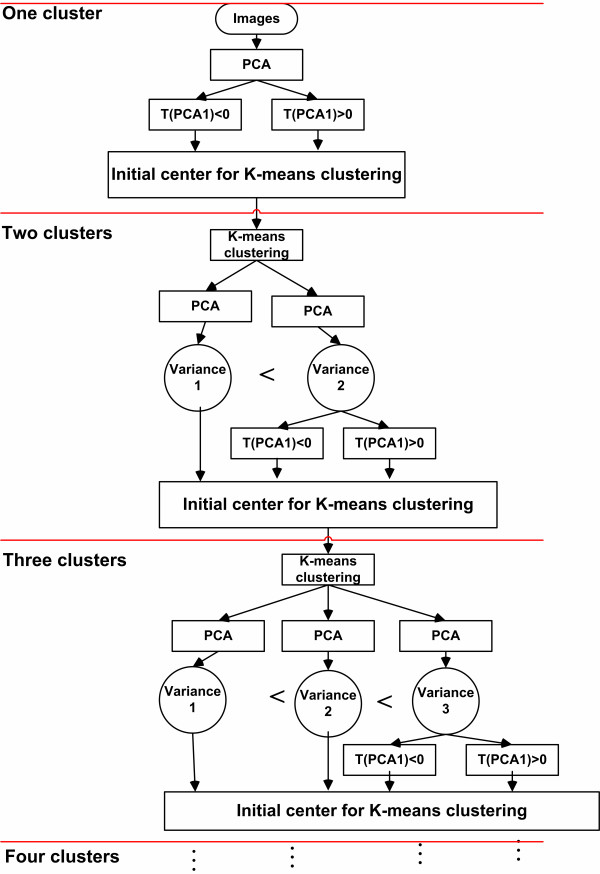
K-means clustering algorithm used for identifying cell regions in fluorescence images.

Principal component analysis can be performed on *X *to determine pixels with similar brightness in the images [12]:

(3) *X *= *TP*^T^+*E*

where *T *is the score matrix, *P *is the loading matrix, and *E *is the residual between the actual image data and the reconstruction by PCA. The columns of *P *represent principle components of the image data matrix, while the columns of *T *are the projections of the image data matrix onto the principle components. An illustration of the data and the first principal component (PC1) is shown in Figure 2. The projection of a point onto PC1 can be used as a measure for clustering the pixel brightness into different sets via K-means clustering. Figure 3 illustrates the procedure of fluorescent cell searching based on K-mean clustering and PCA. In an initial step PCA is used to divide the pixels of the image into two clusters based upon their projection onto PC1. K-means clustering iteratively updates the pixels and centroids of the two clusters until the sum of distances from all the pixels in each cluster is minimized. The cluster with the larger variation is divided in a next step. The centroids of the two new clusters, which are determined by PCA, and the centroid of the un-divided cluster are used as the initial centroids of the three clusters for K-means clustering, which then sorts the pixels of the image belonging to one of the three clusters. This procedure can be repeated until any number of desired clusters is obtained. The clusters with higher fluorescent intensity are considered to represent the cells which show a significant level of fluorescence. Once the cell region has been determined it is possible to compute the average fluorescence intensity by the following formula:

(4) $$I={\left(\frac{\sum_{k=1}^{N_{f}}I_{f,k}}{N_{f}}-\frac{\sum_{k=1}^{N_{b}}I_{b,k}}{N_{b}}\right)}_{stimulation}$$

*I*_*f*,*k *_refers the fluorescent intensity of the *k*_th _pixel in a fluorescent cell region, *I*_*b*,*k *_refers the fluorescent intensity of the *k*_th _pixel belonging to the background, *N*_*f *_is the total number of pixels in the fluorescent cell region, *N*_*b *_is the total number of pixels in the background. For a RGB image, the fluorescent intensity *I *is defined as the sum of the values of red and green and blue of each pixel. The reason for subtracting the intensity of the pixels representing the background is to reduce measurement noise due to brightness variations.

**Figure 4:**
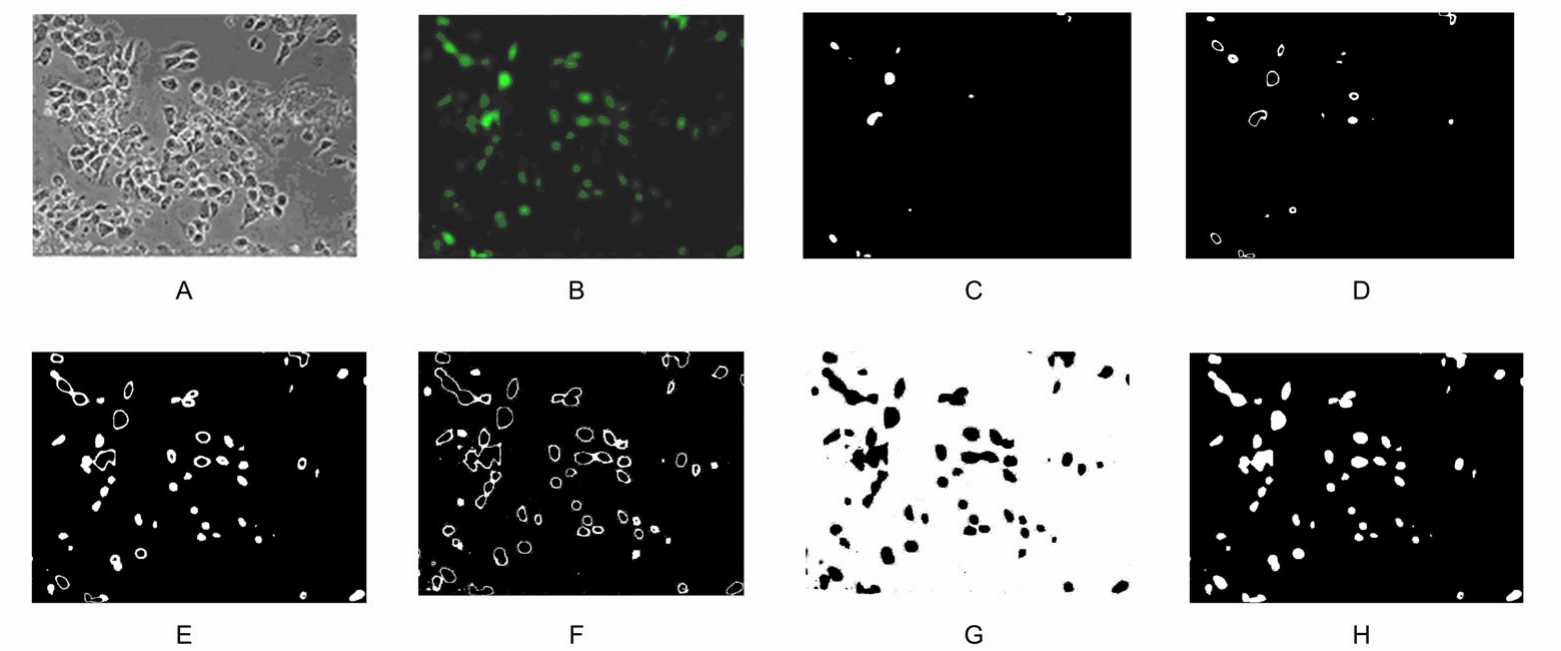
**Results of the image analysis algorithm.** (A) Fluorescence microscopy image, (B) Fluorescent regions detected by the image analysis procedure: (C) – (G) clusters 1 through 5 detected by the algorithm; white pixels refer to pixels included in a specific cluster, (H) cumulative results of clusters 1, 2, and 3; the white region in (H) is chosen as the region representing cells with GFP while the black pixels shown in (H) represent the background.

This procedure has to be repeated for each image taken at different points in time to generate a time series of data for the fluorescence intensity. An example of the outcome of this procedure can be seen in Figure 4 where the first three clusters represent fluorescent cells while the pixels included in clusters 4 and 5 corresponds to the background.

### Model Development

**Figure 5:**
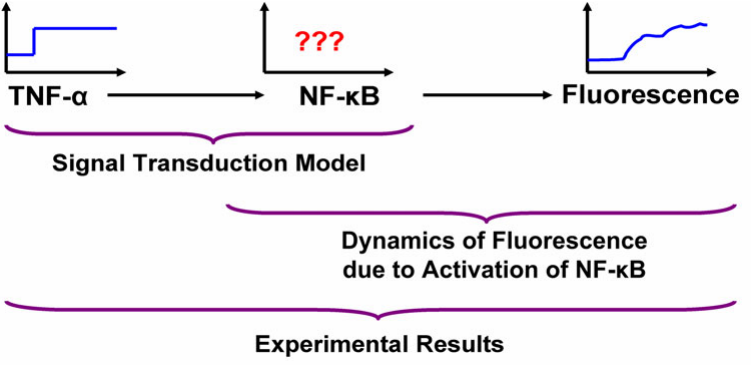
Relationship between input, output, and concentration of transcription factors with GFP-reporter systems.

Two models are involved in this work: (a) a model describing the dynamics of the proteins involved in TNF-α signaling and (b) a model describing the dynamics of the proteins of a green fluorescent protein reporter system. The first model has the TNF-α concentration as the input to the system and the output of the system is the dynamic profile of NF-κB that results from TNF-α stimulation. The second model uses the NF-κB concentration as the input and predicts the fluorescence intensity profile that can be measured. Using these two models it is possible to determine the NF-κB concentration during an experiment by solving an inverse problem of the second model. The generated data set can then be further used to adjust parameters of the first model. Figure 5 illustrates the relationship between these two models.

**Figure 6:**
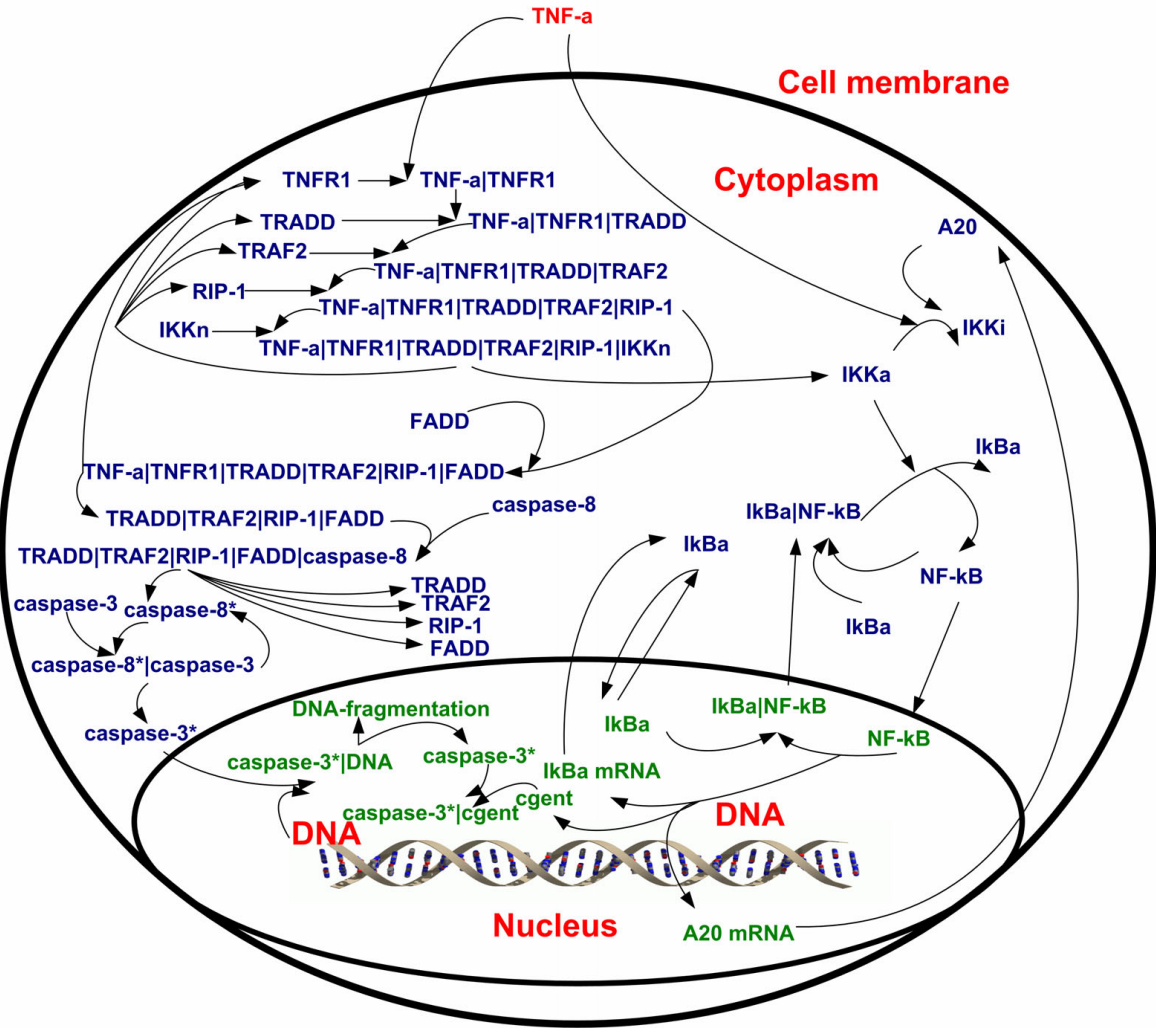
TNF-α signaling pathway.

The model describing TNF-α mediated signal transduction is shown in Figure 6 and the equations are given in 'Additional file 1'. This model is based upon the models described by Rangamani and Sirovich [10] and Lipniacki et al. [11]. The model from Lipniacki et al. was used to describe signal transduction from IKKn to NF-κB whereas the model from Rangamani and Sirovich's work was used to describe signal transduction from TNF-α to IKKn. The reason for combining these two models is that the model from Lipniacki et al.'s work does not describe signal transduction from TNF-α to IKKn, while the paper by Rangamani and Sirovich states that the signal transduction from IKKn to NF-κB as described in their model should be updated as it represents a simplification of what is currently known about the signal transduction pathway. In order to combine these two models the assumption that c-IAP in the reaction "Caspase-3*+c-IAP->caspase-3*|c-IAP" from Rangamani and Sirovich's model can be replaced with cgen_t _from Lipniacki et al.'s model. The rationale behind this assumption is that c-IAP and cgen_t _are both involved in transcription of DNA.

This integrated model, which consists of 37 differential equations and 60 parameters, can represent the dynamic behavior of the proteins involved in TNF-α-mediated NF-κB activation: TNF-α initiates the signal transduction by binding to its receptor TNFR1 and forming the complex TNF-α|TNFR1, which then recruits TRADD, TRAF2, RIP-1 to form the complex TNF-α|TNFR1|TRADD| TRAF2|RIP-1. This complex then activates two pathways: 1) it activates the apoptotic machinery by recruiting FADD; 2) it activates the NF-κB pathway by promoting the neutral form of IKK (IKKn) to the active form of IKK (IKKa). NF-κB is then released from the complex NF-κB|IκBα and translocates into the nucleus to initiate the transcription/translation process. Since the presence of NF-κB in the nucleus (i.e., activation of NF-κB) does not immediately lead to fluorescence seen in the images it is required to augment the developed model with equation describing transcription/translation as well as activation of GFP. The equations to be added are based upon the model described by Subramanian and Srienc [9] where modifications are made to account for the constant reporter DNA levels in our experiments (i.e., due to stable integration of the reporter plasmid into the genomic DNA in our reporter cell line [7]) as well as to include the effect of transcription factor concentrations on the transcription rate. These changes result in the following model describing the measurement dynamics:

(5) $$\begin{array}{l} \frac{dm}{dt}=S_{m}p\frac{C_{NF-\kappa B}}{C+C_{NF-\kappa B}}-D_{m}m\\ \frac{dn}{dt}=S_{n}m-D_{n}n-S_{f}n\\ \frac{df}{dt}=S_{f}n-D_{n}f \end{array}$$

where *C*_NF-κB _is the concentration of activated NF-κB in the nucleus, *m *is the mRNA concentration, *n *is the concentration of GFP, and *f *corresponds to the concentration of activated GFP. The values of the parameters shown in equation (5) are given in Table 1. The procedure for estimation of *C *is described below.

**Table 1:** Parameters for the model shown in equation (5).

Parameter	Value	Parameter	Value
*S*_m_	373 1/hr	*S*_*f*_	0.347 1/hr
*D*_m_	0.45 1/hr	*C*	108 nM
*S*_n_	780 1/hr	*P*	5 nM
*D*_n_	0.5 1/hr	*m*(0), *n*(0), *f*(0)	0 nM

The experimental measurements consist of the fluorescence intensity, *I*, as seen on the images which is directly proportional to the concentration of activated green fluorescent protein:

(6) *f *= Δ*I*

where Δ is the ratio between activated GFP and computed fluorescence intensity.

As *I *can be obtained from the fluorescence images that have been processed by the procedures described in the image analysis section, the dynamics of NF-κB can be computed by solving an inverse problem involving equations (5).

## Results

**Figure 7:**
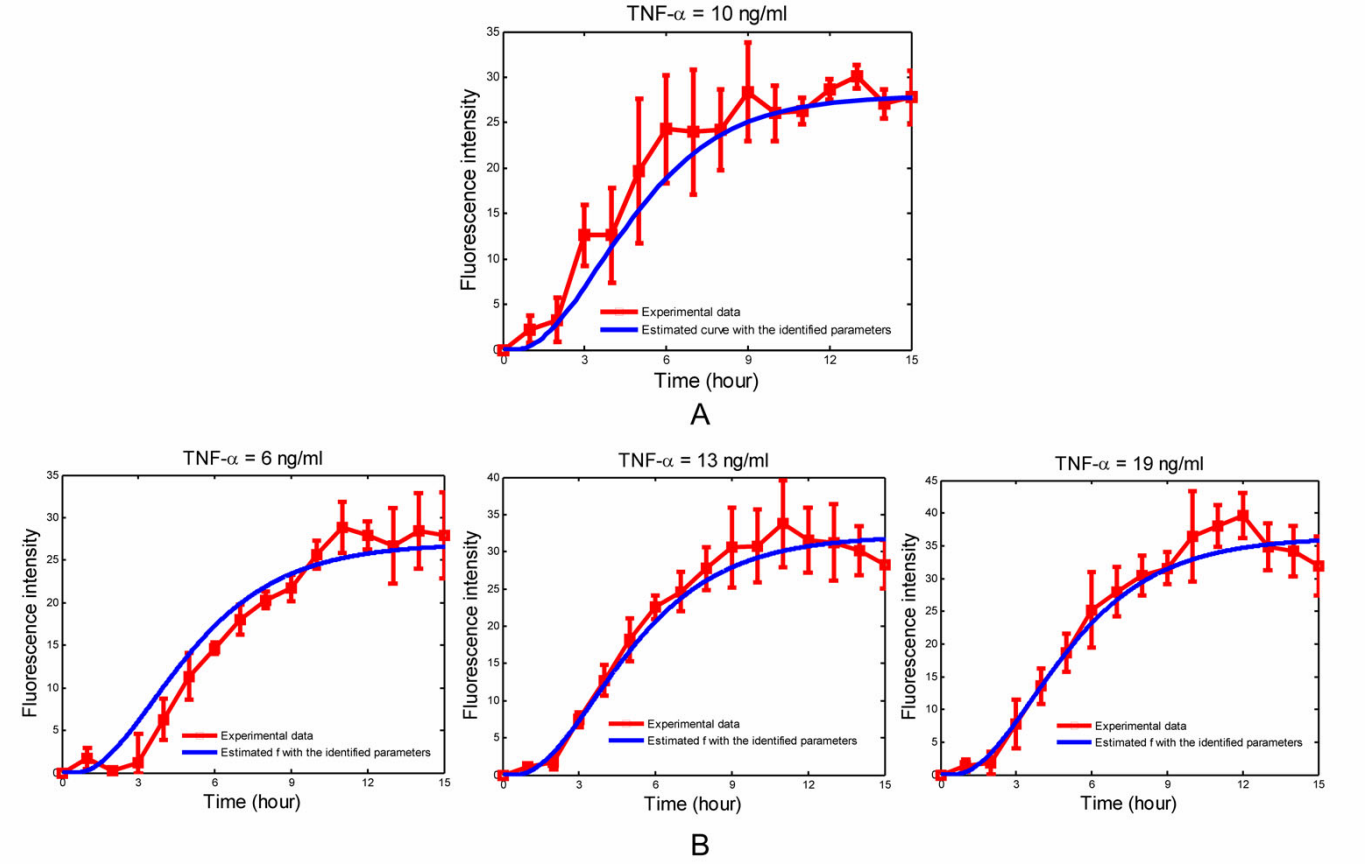
**(A) Comparison of experimental data and the model predictions for *f*/Δ where the NF-κB concentration serves as the input to the model and is taken from Hoffman et al.'s paper**[13]; **(B) Experimental data and the fitted curve *f*/Δ for different TNF-α concentrations.**

The activation of NF-κB in H35 reporter cells was investigated by stimulating with different TNF-α concentrations (6 ng/ml, 10 ng/ml, 13 ng/ml, and 19 ng/ml) as described in the Methods section. The data was analyzed using the described image analysis procedure, resulting in the fluorescence intensity profiles shown (red line) in Figure 7. The error bars indicated +/- one standard deviation from the mean of the measurements taken for each time point.

We developed a procedure that computes the NF-κB concentration profile from the experimental data by solving an inverse problem given by equations (5) and (6). In order to avoid a numerical solution of this inverse problem, we derived an analytical solution which computes *C*_NF-κB _from the fluorescence intensity profile *I*. This analytical solution treats equation (5) as a static nonlinearity

(7) $$u=\frac{C_{\text{NF}-\kappa \text{B}}}{C+C_{\text{NF}-\kappa \text{B}}}$$

which is followed by a system of linear differential equations:

(8) $$\begin{array}{c} dm/dt=S_{m}pu-D_{m}m\\ dn/dt=S_{n}m-D_{n}n-S_{f}n\\ df/dt=S_{f}n-D_{n}f \end{array}$$

Taking a Laplace transform of equation (8) results in *f*(*s*) as a function of *u*(*s*):

(9) $$f(s)=\frac{S_{f}}{s+D_{n}}\cdot \frac{S_{n}}{s+D_{n}+S_{f}}\cdot \frac{S_{m}p}{s+D_{m}}u(s)$$

While it is possible to choose any function to describe *u*(*s*), we opted for

(10) $$u(s)=\frac{\omega_{n}^{2}}{s^{2}+2\varepsilon \omega_{n}s+\omega_{n}^{2}}\cdot \frac{T_{\alpha }}{s}$$

as *u*(*s*) represents a concentration profile of *C*_NF-κB _that shows damped oscillatory behavior as has been reported in the literature [13]. Substituting equation (10) into equation (9) and performing an inverse Laplace transform results in:

(11) $$f(t)=A_{1}+A_{2}e^{-D_{n}t}+A_{3}e^{-(D_{n}+S_{f})t}+A_{4}e^{-D_{m}t}+A_{7}e^{-\varepsilon \omega_{n}t}\sin(\omega_{n}\sqrt{1-\varepsilon^{2}}t+\varphi )$$

where *A*_1_, *A*_2_, *A*_3_, *A*_4_, *A*_7_, and *ϕ *are constants with the values given in 'Additional file 2'.

The values of the parameters *ε*, *ω*_*n *_and *T*_*α *_are estimated by fitting *f*(*t*) to the experimental data for each experiment. The concentration of NF-κB is then given by:

(12) $$\begin{array}{ccc} C_{\text{NF}-\kappa \text{B}}=\frac{CT_{\alpha }\sqrt{1-\varepsilon^{2}}-CT_{\alpha }e^{-\varepsilon \omega_{n}t}\sin(\omega_{n}\sqrt{1-\varepsilon^{2}}t+\phi )}{\text{(1-}T_{\alpha })\sqrt{1-\varepsilon^{2}}+T_{\alpha }e^{-\varepsilon \omega_{n}t}\sin(\omega_{n}\sqrt{1-\varepsilon^{2}}t+\phi )}, & \text{where} & \phi =\arctan\frac{\sqrt{1-\varepsilon^{2}}}{\varepsilon } \end{array}$$

The values of *C *from equation (7) and Δ from equation (6) only need to be estimated once and can be assumed to be constant for all future experiments. We have chosen the concentration profile for NF-κB as reported in the paper by Hoffman et al. [13], which corresponds to a stimulation with 10 ng/ml of TNF-α, as the input, and have estimated *C *and Δ from experimental data that we have collected for stimulation with 10 ng/ml of TNF-α. The value of *C *was determined to be 108 nM and Δ was found to be equal to 2.5562 × 10^4^. It should be noted that some of the data derived from a stimulation with 10 ng/ml of TNF-α was used for determining these parameter values, while other data points will be used for testing model. Figure 7A shows the fit of equation (11) to the data generated by this experiment.

**Figure 8:**
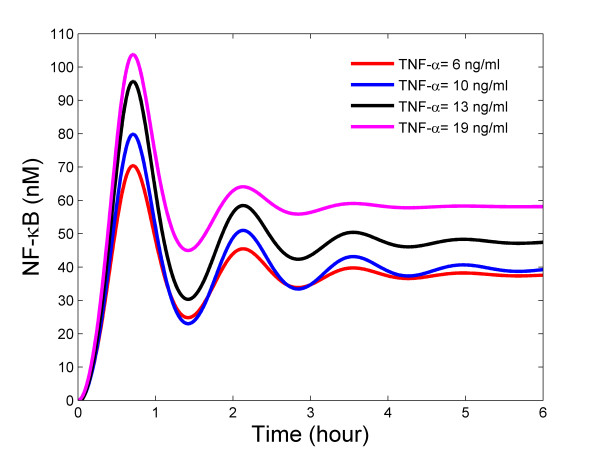
NF-κB profiles computed via solution of the inverse problem for TNF-α concentrations of 6 ng/ml, 10 ng/ml, 13 ng/ml, and 19 ng/ml.

Figure 7B depicts the experimental data for stimulation with 6 ng/ml, 13 ng/ml, and 19 ng/ml of TNF-α as well as the results of the system identification using equation (11). The values for *C *and Δ are constant for these experiments, however, the values for *ε, ω*_*n*_and *T*_*α *_are estimated separately for each data set. The corresponding concentration profiles for NF-κB, as computed by equation (12) are shown in Figure 8. It can be seen that stimulation with higher concentrations of TNF-α results in larger long-term concentrations of NF-κB as well as in higher peak concentrations. One important aspect of this procedure is that the data obtained is quantitative (i.e., numerical values of the NF-κB profile at each time point are obtained) and not merely qualitative.

**Figure 9:**
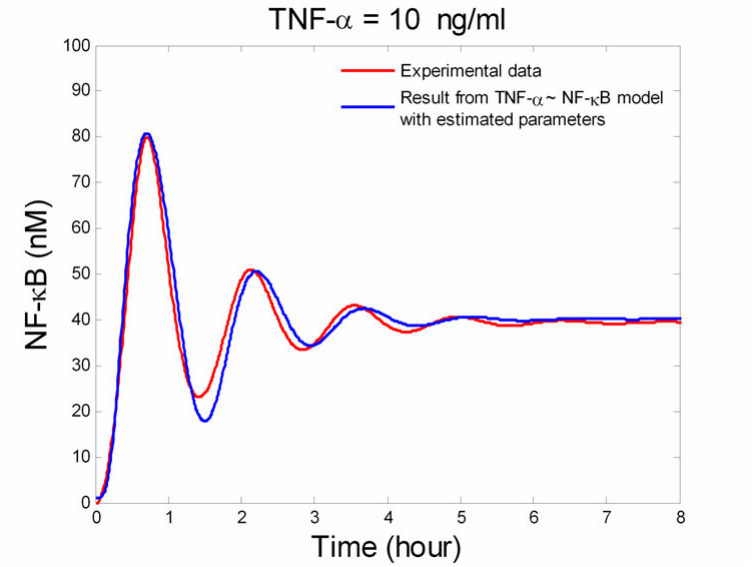
Comparison between NF-κB profiles computed via the presented technique for 10 ng/ml of TNF-α and simulation of the model where some parameters have been re-estimated.

These results for stimulation with 6 ng/ml, 13 ng/ml, and 19 ng/ml of TNF-α were used to estimate parameters of the signal transduction pathway model. Since the developed model contains many more parameters than can be estimated from three time series of data, it was required to use local sensitivity analysis to determine which parameters should be re-estimated. It was determined that the parameters *c*_3_, *k*_1*p*_, and *k*_*r *_are good candidates for estimation. Nonlinear least square routines in MATLAB were then used to estimate these three parameters. The estimated values were found to be 0.0104, 0.0740 and 2.50, respectively. Since the data derived from the stimulation with 10 ng/ml of TNF-α was not used for estimating these parameters, this data set can be used for validating the accuracy of the updated model. Figure 9 shows the model prediction for 10 ng/ml of TNF-α together with the experimental results derived from the described image analysis procedure. It can be concluded that the updated model predicts experimental data very well.

## Discussion

In this study, we have demonstrated that transcription factor activation profiles can be quantitatively extracted from fluorescence reporter data. The proposed approach was effective in deriving transcription factor activation rates from GFP profiles generated from NF-κB reporter cells stimulated with 10 – 50 ng/mL of TNF-α, a concentration range that is commonly used in cell culture experiments [5,14] and reported to result in strong activation of NF-κB [8]. However, predicting NF-κB activation at lower concentrations of TNF-α (< 10 ng/mL) was not as effective due to low levels of GFP signal. This is evident from Figure 7B which shows a better correlation between the model and experimental data at higher (13 and 19 ng/mL) than at lower (6 ng/mL) TNF-α concentrations. Therefore, while our method is effective for moderate-to-high levels of activation, further improvement (e.g., in the image analysis methods) is needed to increase the GFP signal/noise ratio for effectively predicting profiles of low abundance transcription factors.

Another discrepancy between the model and experimental data is predicting long-term NF-κB activation profiles. The data in Figure 7B shows that fluorescence decreases after ~11 h even though the stimulus (TNF-α) is continually present, with the decrease being more pronounced at the higher concentrations. However, this decrease is not reflected in Figure 7B which shows NF-κB levels being constant beyond 11 h as the assumed model structure from equation (10) cannot represent this decrease. It is possible to postulate a different profile for the transcription factor, resulting in differences in equation (10), e.g., one that can reflect such a decrease. However, it is not clear if the decrease in fluorescence observed after ~11 h of stimulation results from experimental artifacts (i.e., fluorescence photobleaching and cell death arising from cells being repeatedly exposed to UV light for imaging) or is a real biological phenomenon (i.e., consequence of change in gene expression arising due to constant stimulation with TNF-α). A better understanding of long-term activation is needed to evaluate this behavior.

It should also be noted that the model describing the activation of NF-κB by TNF-α is not required for deriving NF-κB profiles from GFP profiles. However, use of the 1^st ^principles model enables us to estimate model parameters using the data and thereby refine the model describing activation of NF-κB by TNF-α, so as to develop a systems level understanding of TNF-α signaling. In this paper, we have utilized the fact that a considerable body of literature is present on TNF-α induction of NF-κB activation. Previously developed models and experimental data [13] suggest that NF-κB exhibits oscillatory behavior upon exposure to TNF-α. However, our overall approach for deriving transcription factor activation profiles is also valid for other transcription factors where the activation profile is not well characterized. In such cases, it will be necessary to assume different transcription factor activation profiles and verify the model prediction by comparing the predicted fluorescence intensity profiles with the experimental data.

In summary we have developed a methodology for quantitatively determining transcription factor profiles. This technique makes use of fluorescence microscopy images from a GFP reporter system for transcription factor activation and involves solving an inverse problem to determine the transcription factor profile from the fluorescence intensity dynamics. Data generated by this method can then be used to estimate parameters for signal transduction pathway models. This technique was applied to the activation of NF-κB by TNF-α, however, it can be used to determine transcription factor profiles for any system where limited qualitative knowledge about the transcription factor dynamics exists.

## Authors' contributions

ZH implemented the image analysis procedure as well as devised the solution technique for the model inversion which computes the transcription factor profile. FS performed all NF-κB reporter experiments. JH and AJ supervised the computational and experimental aspects of the work, respectively, and were involved in preparation of the manuscript.

## Supplementary Material

Additional file 1 This file contains the equations, the initial values of state variables, and the parameters of the model describing TNF-α mediated signal transduction. Click here for file

Additional file 2 This file contains the equations for computing the values of the constants found in equation (11). Click here for file
