# Supplementary material for: Integrated modeling and experimental approach for determining transcription factor profiles from fluorescent reporter data
Source: BMC Syst Biol. 2008 Jul 17;2:64. doi: 10.1186/1752-0509-2-64 (PMC2491602; doi:10.1186/1752-0509-2-64)
Supplement: Additional file 1 — This file contains the equations, the initial values of state variables, and the parameters of the model describing TNF-α mediated signal transduction. [file 1752-0509-2-64-S1.doc]

**Additional file 1**

Model equations

State variables of the model and their initial values:

| Name | Species | Initial values (µM) |
| --- | --- | --- |
| *x*_1_ | TNFR1 | 0.1 |
| *x*_2_ | TNF-α/TNFR | 0 |
| *x*_3_ | TRADD | 0.15 |
| *x*_4_ | TNF-α/TNFR1/TRADD | 0 |
| *x*_5_ | TRAF2 | 0.1 |
| *x*_6_ | TNF-α/TNFR1/TRADD/TRAF2 | 0 |
| *x*_7_ | RIP-1 | 0.1 |
| *x*_8_ | TNF-α/TNFR1/TRADD/TRAF2/RIP-1 | 0 |
| *x*_9_ | IKKn | 0.2 |
| *x*_10_ | TNF-α/TNFR1/TRADD/TRAF2/RIP-1/IKKn | 0 |
| *x*_11_ | IKKa | 0 |
| *x*_12_ | inactive IKK | 0 |
| *x*_13_ | cytoplasmic IKK|IkBa complex | 0 |
| *x*_14_ | cytoplasmic IKK|IkBa|NF-κB complex | 0 |
| *x*_15_ | free cxtoplasmic NF-κB | 0.0003 |
| *x*_16_ | free nuclear NF-κB | 0.0023 |
| *x*_17_ | cytoplasmic A20 | 0.0048 |
| *x*_18_ | A20 transcription | 0 |
| *x*_19_ | free cytoplasmic IkBa | 0.0025 |
| *x*_20_ | free nuclear IkBan | 0.0034 |
| *x*_21_ | IkB transcription | 0 |
| *x*_22_ | cytoplasmic IkBa|NF-κB complex | 0.0592 |
| *x*_23_ | Nuclear IkBa|NF-κB complex | 0.0001 |
| *x*_24_ | Control gene mRNA level or c-IAP | 0 |
| *x*_25_ | FADD | 0.1 |
| *x*_26_ | TNF-α/TNFR1/TRADD/TRAF2/RIP-1/FADD | 0 |
| *x*_27_ | TRADD/TRAF2/RIP-1/FADD | 0 |
| *x*_28_ | Caspase-8 | 0.08 |
| *x*_29_ | TRADD/TRAF2/RIP-1/FADD/caspase-8 | 0 |
| *x*_30_ | Caspase-8* | 0 |
| *x*_31_ | Caspase-3 | 0.2 |
| *x*_32_ | Caspase-8*/caspase-3 | 0 |
| *x*_33_ | Caspase-3* | 0 |
| *x*_34_ | DNA-fragmentation | 0 |
| *x*_35_ | Caspase-3*/c-IAP | 0 |
| *x*_36_ | DNA intact | 0.8 |
| *x*_37_ | Caspase-3*/DNA | 0 |

Note: *u* is the concentration of TNF-α, ng/ml. The molecule weight of TNF-α is 17 kDa. The unit ng/ml can be converted to µM by dividing by 17×10^3^. *y* is the system output NF-κB after being scaled by *k_r_* in units of µM.

Values of the parameters

| Name | Value | Name | Value |
| --- | --- | --- | --- |
| *k*_v_ | 5 | *k*_1p_ | 0.0740 (0.185) |
| *AB*^*^ | 1 | *k*_15p_ | 0.185 |
| *c*_1_ | 5×10^-7^ *AB* | *k*_2p_ | 0.00125 |
| *c*_2_ | 0 | *k*_16p_ | 0.00125 |
| *c*_3_ | 0.0104 (0.0004) | *k*_3p_ | 0.185 |
| *c*_4_ | 0.5 | *k*_17p_ | 0.37 |
| *c*_5_ | 0.0003 | *k*_4p_ | 0.00125 |
| *k*_1_ | 0.0025 | *k*_18p_ | 0.5 |
| *k*_2_ | 0.1 | *k*_5p_ | 0.185 |
| *k*_3_ | 0.0015 | *k*_19p_ | 0.2 |
| *k*_deg_ | 0.000125 | *k*_6p_ | 0.00125 |
| *a*_2_ | 0.2 | *k*_20p_ | 0.1 |
| *a*_1_ | 0.5 | *k*_7p_ | 0.185 |
| *a*_3_ | 1. | *k*_21p_ | 0.1 |
| *t*_1_ | 0.1 | *k*_8p_ | 0.00125 |
| *t*_2_ | 0.1 | *k*_22p_ | 0.06 |
| *AA*^*^ | 1 | *k*_9p_ | 0.185 |
| *c*_1a_ | 5×10^-7^ *AA* | *k*_23p_ | 100 |
| *c*_2a_ | 0 | *k*_10p_ | 0.00125 |
| *c*_3a_ | 0.0004 | *k*_24p_ | 0.185 |
| *c*_4a_ | 0.5 | *k*_11p_ | 0.37 |
| *c*_5a_ | 0.0001 | *k*_25p_ | 0.00125 |
| *c*_6a_ | 0.00002 | *k*_12p_ | 0.014 |
| *i*_1_ | 0.0025 | *k*_26p_ | 0.37 |
| *e*_2a_ | 0.01 | *k*_13p_ | 0.00125 |
| *i*_1a_ | 0.001 | *k*_14p_ | 0.37 |
| *e*_1a_ | 0.0005 | *k*_28p_ | 0.5 |
| *c*_1c_ | 5×10^-7^ | *p* | 1.75 |
| *c*_2c_ | 0 | *T*_r_^*^ | 1 |
| *c*_3c_ | 0.0004 | *k*_r_ | 2.5 |

* Note: 1) *AA* = 1 refers to wt cell, while *AA* = 0 refers to IkBa deficient cell

2) *AB* = 1 refers to wt cell, while *AB* = 0 refers to A20 deficient cell

3) *T*_r_ = 0 when TNF-α is off, while *T*_r_ = 1 when TNF-α is on

4) Values in brackets refer to the model fit to the experimental data
