# Supplementary material for: Integrated modeling and experimental approach for determining transcription factor profiles from fluorescent reporter data
Source: BMC Syst Biol. 2008 Jul 17;2:64. doi: 10.1186/1752-0509-2-64 (PMC2491602; doi:10.1186/1752-0509-2-64)
Supplement: Additional file 2 — This file contains the equations for computing the values of the constants found in equation (11). [file 1752-0509-2-64-S2.doc]

**Additional file 2**

Equations for computing the values of the constants found in equation (11)

$$\begin{matrix} A_{1}=S_{f}S_{n}S_{m}pT_{a}/D_{n}/(D_{n}+S_{f})/D_{m} \\ A_{2}=-S_{n}S_{m}p\omega_{n}^{2}T_{a}/(D_{m}-D_{n})/(D_{n}^{2}-2\varepsilon\omega_{n}D_{n}+\omega_{n}^{2})/D_{n} \\ A_{3}=S_{n}S_{m}p\omega_{n}^{2}T_{a}/(D_{m}-D_{n}-S_{f})/((D_{n}+S_{f})^{2}-2\varepsilon\omega_{n}(D_{n}+S_{f})+\omega_{n}^{2})/(D_{n}+S_{f}) \\ A_{4}=S_{f}S_{n}S_{m}p\omega_{n}^{2}T_{a}/(D_{n}-D_{m})/(D_{m}-D_{n}-S_{f})/(D_{m}^{2}-2\varepsilon\omega_{n}D_{m}+\omega_{n}^{2})/D_{m} \\ A_{5}=-C_{0}d_{1}/(bd_{1}^{2}+bd_{0}^{2}) \\ A_{6}=C_{0}(ad_{1}+bd_{0})/(bd_{1}^{2}+bd_{0}^{2}) \\ A_{7}=\sqrt{A_{5}^{2}+(\frac{A_{6}-A_{5}\varepsilon\omega_{n}}{\omega_{n}\sqrt{1-\varepsilon^{2}}})^{2}} \\ a=-\varepsilon\omega_{n} \\ b=\omega_{n}\sqrt{1-\varepsilon^{2}} \\ d_{1}=-(a_{3}+4a)b^{3}+(3a_{3}a^{2}+2a_{2}a+4a^{3}+a_{1})b \\ d_{0}=b^{4}+a_{3}a^{3}-(3a_{3}a+a_{2}+6a^{2})b^{2}+a_{2}a^{2}+a^{4}+a_{1}a \\ a_{1}=(D_{n}^{2}+D_{n}S_{f})D_{m} \\ a_{2}=D_{n}^{2}+D_{n}S_{f}+2D_{n}D_{m}+D_{m}S_{f} \end{matrix}$$

$$\begin{matrix} a_{3}=2D_{n}+D_{m}+S_{f} \\ C_{0}=S_{f}S_{n}S_{m}p\omega_{_{n}}^{2}T_{\alpha} \\ \phi=\arctan\frac{A_{5}\omega_{n}\sqrt{1-\varepsilon^{2}}}{A_{6}-A_{5}\varepsilon\omega_{n}} \end{matrix}$$
